# Childhood trauma in adult depressive and anxiety disorders: immuno-metabolic evidences in the NESDA cohort

**DOI:** 10.1192/j.eurpsy.2024.51

**Published:** 2024-08-27

**Authors:** Y. Milaneschi

**Affiliations:** Amsterdam UMC, Amsterdam, Netherlands

## Abstract

**Abstract:**

Childhood trauma and depression are both associated with increased risk of metabolic disorders, but their joint effects and underlying mechanisms are not well understood. This talk will present recent findings from large-scale epidemiological and biobank studies that explore the metabolic signature of childhood trauma, depression, and their interplay. For example, using longitudinal data from the NESDA cohort, we investigated the association of childhood trauma with metabolic syndrome in ˜3000 adults, including patients with depression and/or anxiety and healthy controls, over 9 years of follow-up. The talk will also describe preliminary results from an individual patient data meta-analysis pooling >160,000 subjects from the Early Cause European Consortium. In this study, we examined the differences in markers of obesity and dyslipidemia across individuals with neither childhood trauma nor depression (controls), those with childhood maltreatment, those with depression, those reporting both of these conditions. The findings described in the talk shed light on the complex interplay between early life stress, mood disorders, and metabolic health.

**Disclosure of Interest:**

None Declared

